# Supplementary material for: Analysis of the Model of Atherosclerosis Formation in Pig Hearts as a Result of Impaired Activity of DNA Repair Enzymes
Source: Int J Mol Sci. 2024 Feb 14;25(4):2282. doi: 10.3390/ijms25042282 (PMC10888614; doi:10.3390/ijms25042282)
Supplement: Supplementary file 1 [file ijms-25-02282-s001.zip › ijms-2785352-supplementary.pdf]

Table S1. Data published previously [41, 82]. Ultrasound measurements of the thickness of complex intima+media of the femoral artery (IMT FA), High-density lipoprotein (HDL), low-density lipoprotein (LDL), triglycerides (TG). * Statistical significance.

	**BDG**		**UDG**		**RG**	
	**Start**	**end**	**start**	**End**	**Start**	**end**
IMT FA ultrasound (μm)		638±87		705±108*		686±102
Glucose (mg/dl)	87.7±14.42	94.6±13.7	96.8±18.7	102.5±27.4	100.3±22	103.8±26.1
Insulin (μg/ml)	38.8±4.78	45.7±14.65	42.3±11.6	52.8±19.7	53.3±30	79.4±52.9
Cholesterol serum (mmol/l)	-	2.1±0.45	-	2.02±0.48	-	1.9±0.51
HDL serum (mmol/l)	-	0.75±0.13	-	0.88±0.34	-	0.65±0.1*
LDL serum (mmol/l)	-	1.09±0.24		1.17±0.17	-	1.18±0.2
TG serum (mmol/l)	-	0.32±0.2	-	0.53±0.34	-	0.49±0.21*
